# Effects of dengue virus type-2 serotype (DENV-2) on the expression profile of matrix metalloproteinases in THP-1, monocyte cells and their roles in endothelial dysfunctions: protective effect of atorvastatin

**DOI:** 10.3389/fcimb.2025.1632634

**Published:** 2025-10-20

**Authors:** Rituraj Niranjan, Pitchavel Vidhyapriya, Vyshali Murugasamy, Subramanian Muthukumaravel, Ashwani Kumar

**Affiliations:** ^1^ Division of immunology, ICMR-National Institute of Research in Tribal Health, Department of Health Research, Ministry of Health and Family Welfare, Government of India, Jabalpur, M.P, India; ^2^ Immunology Laboratory, Division of Microbiology and Immunology, Indian Council of Medical Research (ICMR)-Vector Control Research Centre, Puducherry, India; ^3^ Faculty of Academy of Scientific and Innovative Research, Ghaziabad, Uttar Pradesh, India; ^4^ Indian Council of Medical Research (ICMR)-Vector Control Research Centre, Puducherry, India

**Keywords:** dengue virus, matrix metalloproteinases (MMPs), apoptosis, angiogenesis, monocyte, atorvastatin

## Abstract

Dengue viral fever is one of the most important arboviral infections, particularly in tropical and subtropical regions. In a dengue infection, monocyte-mediated matrix metalloproteases are speculated to be implicated in tissue damage and vascular leakage. However, the exact mechanisms are largely unknown. In the present study, we investigated the expression profiles of MMPs in primary monocytes and in THP-1 cells infected with all dengue virus serotypes. The mechanism of MMP-mediated anti-viral effect of atorvastatin was also investigated in detail on dengue virus-induced expression profiles of mRNA and VEGF. We found elevated mRNA expressions profiles of MMP-2, MMP-9, and MMP-14 in DENV-infected THP-1 cells compared to the uninfected control group. Interestingly, these upregulated expressions of MMPs were reversed by atorvastatin. Similar patterns of mRNA expressions were also observed of MMPs and VEGF members in NS1-injected mice. Atorvastatin downregulated the MMP and VEGF expressions in this NS1-injected mouse model. Next, to prove the role of immune cells in causing endothelial dysfunctions, secretome obtained from dengue virus-induced monocytes was exposed to endothelial cells. Interestingly, this secretome has an elevated expression of pro-apoptotic and angiogenic markers like caspases, angiopoietins, VEGF, and their receptor genes in endothelial (HUVEC) cells. These changes were reversed by atorvastatin in a dose-dependent manner. Furthermore, the mechanistic role of MMP-9 in causing apoptosis and angiogenesis in endothelial cells was established. Thus, we suggested that DENV-2 might cause monocyte-mediated angiogenesis and apoptosis, making endothelial dysfunctions which may resemble the mechanism of pathogenesis of dengue shock syndrome (DHF/DSS). Additionally, our finding shows that atorvastatin has MMPs’ inhibitory potential against dengue, which may be adopted in clinical trials against severe dengue viral disease. The current findings are interesting; however, further studies may be needed to adopt the current findings in the future.

## 1 Introduction

Dengue virus (DENV) is one of the most important arboviral infections, particularly in tropical and subtropical regions. According to estimates by the World Health Organization (WHO), approximately 3.6 billion people worldwide are at risk of dengue infection. Among those at risk, it is estimated that there are about 390 million dengue infections each year (Murugesan and Manoharan, 2020). It is estimated to affect more than 125 countries worldwide (Murray et al., 2013). Dengue virus is a spherical virus enclosed in a lipid envelope; its genome is composed of a positive-sense, single-stranded RNA molecule (Rigau-Pérez et al., 1998). The dengue virus genome codes for three structural proteins (involved in the formation and organization of the virus particle) and seven non-structural proteins (responsible for various aspects of viral replication) (Chambers et al., 1990; Lindenbach et al., 2013). Dengue fever is a viral infection caused a mosquito bite (Fever, 1977). The dengue virus is primarily transmitted to humans through the bite of *Aedes* mosquitoes, particularly *Aedes aegypti* (Banerjee et al., 2020). These mosquitoes are commonly found in semi-urban and urban areas and are active during the day. Dengue viral infections can manifest in a spectrum of clinical presentations, ranging from asymptomatic cases to severe and potentially life-threatening conditions (Guzman et al., 2010). Dengue fever can cause a wide range of symptoms, which, in severe cases, also show mild bleeding manifestations. In some cases, it can progress to a severe and potentially life-threatening form known as dengue shock syndrome (DSS) or dengue hemorrhagic fever (DHF) (Murray et al., 2013; Organization WH, 2022).

The incidence of dengue fever has been increasing in recent decades partly due to factors such as urbanization, globalization, and climate change, which have contributed to the expansion of mosquito populations and the spread of the virus to new areas (Gubler, 2011). It is important to note that while dengue fever is a significant public health concern, there are ongoing efforts to develop vaccines and improve diagnostics and treatment options to combat this disease (Organization WH, 2022).

Innate immune cells play a crucial role in the pathogenesis of dengue viral disease, but the exact mechanisms and contributions of different cell types are still not fully understood. The innate immune response of the body is responsible for the initial recognition and containment of the dengue virus. Several types of innate immune cells are involved in response to dengue virus infection, which include monocytes or macrophages natural killer (NK) cells, neutrophils, and dendritic cells (Fernandes-Santos and Azeredo, 2022). In the host response to dengue virus (DENV) infection, an essential role is played by dendritic cells, and the initial interaction between DENV and the immune system often occurs with dendritic cells located in the dermis (Wu et al., 2000).

In severe dengue disease, the hyper-activated immune system results in increased vascular permeability, endothelial dysfunction, and the release of inflammatory mediators, which contribute to plasma leakage, organ damage, and the development of severe symptoms (Orsi et al., 2014). Understanding the complex interactions between the dengue virus and the innate immune system is crucial to develop effective therapeutic interventions and vaccines.

Various vascular tissues and immune cells express matrix metalloproteinases (MMPs) (Itoh, 2006). Matrix metalloproteases are a family of enzymes involved in the remodeling of various tissues, including extracellular matrix, which is the structural framework of tissues. These enzymes play important roles in tissue repair, angiogenesis, and inflammation (Almalki et al., 2017). However, when MMPs are deregulated or overactive, they can contribute to various pathological conditions, including cancer and neurological disorders (Birkedal-Hansen et al., 1993; Singh et al., 2015). In dengue infection, matrix metalloproteases may be involved in tissue damage and vascular leakage observed in severe dengue cases. MMP-2 and MMP-9 have been implicated in the breakdown of the basement membrane and extracellular matrix components, contributing to vascular permeability and leakage observed in severe dengue infections (van de Weg et al., 2014). MMP-2 has also been associated with the activation of pro-inflammatory cytokines and chemokines, promoting inflammation in dengue. Additionally, MMP-8, also known as collagenase-2, is involved in tissue remodeling and the degradation of collagen, a major component of the extracellular matrix. MMP-8 has been suggested to contribute to tissue damage and inflammation in dengue. However, the exact contributions of specific matrix metalloproteases and their regulation in dengue pathogenicity are not yet well defined (van de Weg et al., 2014).

The combined effects of DENV infection and the action of inflammatory and angiogenesis mediators on the infected endothelium result in augmented angiogenic changes and macromolecule permeability (Liu et al., 2009). Understanding these mechanisms is important to elucidate the pathogenesis of severe dengue and develop strategies to mitigate the vascular and inflammatory manifestations of the disease. On the other hand, some drugs including statins have indicated some inhibition in dengue virus replication. However, their mechanism associated to immune cell-mediated MMP actions is still unelucidated (Bryan-Marrugo et al., 2016; Allela et al., 2024; Jani et al., 2025). In a study, it was shown that atorvastatin has shown antiviral effect on dengue virus production via inhibiting the virus assembly (Martinez-Gutierrez et al., 2011; Whitehorn et al., 2012).

Apoptosis is known to play a key role in cell death in response to dengue virus (DENV) infection in various cell types, including human neuronal cells, liver cells, and endothelial cells, both *in vitro* and *in vivo*. This suggests that apoptotic cell death is indeed an important feature of DENV pathogenesis. The mechanisms by which dengue virus (DENV) induces apoptosis are not fully understood and can differ between various cell types or tissues. Viruses often interact with host cells in complex and intricate ways, leading to diverse responses based on the specific viral strain, the infected cell type, and the host’s immune response (Courageot et al., 2003).

Along with matrix metalloproteases (also known as matrixins), neutrophils, monocytes, dendritic cells, and epithelial cells are involved in the pathogenicity of dengue (Her et al., 2017; Niranjan et al., 2019b). However, the precise functions of these components in the context of dengue infection still need to be thoroughly understood and are the subject of ongoing research. In the present study, we focus on understanding the regulation and activity of specific MMPs in THP-1 cells exposed to dengue virus type-2 serotype with or without atorvastatin. Along with MMP expression, we have also checked the angiopoietin and apoptotic markers.

## 2 Materials and methods

### 2.1 Cell line culture and maintenance

The THP-1 cell line was obtained from NCCS, Pune, India, and since then was successfully cultured in the tissue culture laboratory of National Institute of Research in Tribal Health (NIRTH). HUVEC cells were procured from HI media. The THP-1 cell line was maintained in RPMI 1640 or Roswell Park Memorial Institute-1640 medium containing 10% fetal bovine serum at 37°C with 5% CO_2_. The cells are always passaged when they reach 90% to 100% of confluency.

### 2.2 Exposure of THP-1 cells with DENV type 2 serotype in the presence of atorvastatin

The THP-1 cells were treated with atorvastatin 30 min before DENV-2 serotype as group-I, and in another group a combination of DENV2+ atorvastatin was used, and only DENV-2-exposed to THP-1 was planned as a different set of groups, and only THP-1 without infection was used as the control. After 24 h of incubation, supernatant was collected, and cells were harvested. Similarly, exposure of purified MMP-9 (procured from R&D Biological Company) protein on the HUVEC/endothelial cells was done, and assessment of angiogenic and apoptosis mediators was performed.

### 2.3 RNA isolation and quantification

The harvested cells were lysed by adding 500 µL of Tri Reagent (TRIzol, Sigma Aldrich), and RNA was isolated by the TRIzol method. Briefly, cells were lysed in TRIzol, and 100 µL of chloroform was added. The mixture was vortexed for 15 s, centrifuged at 12,000*g* for 15 min at 4°C. An aqueous layer was obtained, and 250 µL of iso-propanol was added. It was mixed and incubated for 10 min at 4°C and then spinned at 12,000*g* for 10 min at 4°C. The pellet was washed with 0.5 mL of 75% ethanol and centrifuged at 7,500*g* for 7 min at 4°C. The RNA pellet was dissolved in 20 μL of RNase-free water. RNA quantification was done by using Nanodrop, and its integrity was checked on 1.2% agarose gel.

### 2.4 Synthesis of complementary DNA

cDNA was synthesized from 1 µg of total RNA by using RevertAid First Strand cDNA Synthesis Kit according to the manufacturer’s instructions (Thermo Scientific) and stored at -20°C for further use.

### 2.5 Traditional reverse transcriptase PCR or real-time PCR for the expressional analysis of genes

To understand the mRNA expression profile of MMPs, apoptotic marker, and angiopoietin genes, real-time PCR was done. To accomplish this, cDNA (100 ng) was used as a starting template antisense strand. This was then mixed with 1 µL of primers (0.5 pmol) (**Table 1**), 10 µL of asymmetrical cyanine SYBR green, 3 µL of autoclaved DNase, and RNase-free water. The ΔΔCt method was used for the relative expression of the genes normalized with the reference gene controls (Livak and Schmittgen, 2001). After the PCR was run, the amplicon was analyzed using the agarose gel electrophoresis. Then, 1.2% to 2% of agarose gels was used. Ethidium bromide dye was used to stain the DNA bands. Conventional reverse transcriptase PCR was done as per the protocol described previously by Niranjan et al. (2022) (Niranjan et al., 2022). The details of primers used are provided in the **Tables 1**, **2**.

**Table 1:** Details of the human primers.

Primers	Sequence	Tm
PROX-1	Forward: 5′ CCT ATC CAT TTC AGA GCC CA 3′	58
	Reverse: 5′ TCG GAC TTT ATG AGC GAC AA 3′	56
VEGFA	Forward: 5′ CCT CCG AAA CCA TGA ACT TT 3′	56
	Reverse: 5′ TTC TTT GGT CTG CAT TCA CAT T 3′	57
VEGFC	Forward: 5′ GCC AAC CTC AAC TCA AGG AC 3′	60
	Reverse: 5′ CCC ACA TCT GTA GAC GGA CA 3′	60
VEGFD	Forward: 5′ ATG GAC TCT CGC TCA GCA TC 3′	60
	Reverse: 5′ ATC GGA ACA CGT CCA CAC AA 3′	58
VEGFR3	Forward: 5′ CTG GAGG GAA AAG TCT GG 3′	60
	Reverse: 5′ GTC TTG ATG TCT GCG TGG G 3′	59
VEGFR2	Forward: 5′ CCA GTC AGA GAC CCA CGT TT 3′	60
	Reverse: 5′ AGT CTT TGC CAT CCT GC GA 3′	58
Ang-1	Forward: 5′ TTC CTT TCC TTT GCT TTC CTC 3′	57
	Reverse: 5′ CTG CAG AGC GTT TGT GTT GT 3′	58
Ang-2	Forward: 5′ AAC ATC CCA GTC CAC CTG AG3′	60
	Reverse: 5′ AAC ATC CCA GTC CAC CTG AG 3′	58
GAPDH	Forward: 5′ TCA ACG GAT TTG GTC GTA TTG GG 3′	63
	Reverse: 5′ TGA TTT TGG AGG GAT CCT GC 3′	58
Caspase-3	Forward: 5′ GAG CTG CCT GTA ACT TG 3′	52
	Reverse: 5′ ACC TTT AGA ACA TTT CCA CT 3′	52
Caspase-6	Forward: 5′ ACT GGC TTG TTC AAA GG 3′	50
	Reverse: 5′ CAG CGT GTA AAC GGA G	51
BCL2	Forward: 5′ AGG AAG TGA ACA TTT CGG TGA C 3′	60
	Reverse: 5′ GCT CAG TTC CAG GAC CAG GC 3′	65
BAX	Forward: 5′ TGC TTC AGG GTT TCA TCC AG 3′	58
	Reverse: 5′ GGC AAT CAT CCT CTG 3′	58
BAD	Forward: 5′ GAG TGA GCA GGA AGA CTC CAG C 3′	66
	Reverse: 5′ TCC ACA AAC TCG TCA CTC ATC C 3′	62
DAPK1	Forward: 5′ CAG TGT TGC TCT AGG AAG 3′	59
	Reverse: 5′ GGG ACT GCC ACA AAT GAT GAG C	64

**Table 2:** Details of the mice primers.

Primers	Sequence	Tm
VEGFA	Forward: 5′ ACA CGG TGG TGG AAG AAG AG 3′	60
	Reverse: 5′ CAA GTC TCC TGG GGA CAG AA 3′	60
VEGFC	Forward: 5′ CTA CAG ATG TGG GGG TTG CT3′	60
	Reverse: 5′ GAT TGG CAA AAC TGA TTG TGA C3′	58
VEGFD	Forward: 5′ GAG GCT GCT GCA ACG AAG A3′	59
	Reverse: 5′ GCA CTT ACA ACC CGT ATG GTT3′	59
VEGFR1	Forward: 5′ CTG GAC TGA GAC CAA GCC CAA G3′	64
	Reverse: 5′ GCT CAG ATT CAT CGT CCT GCA C3′	66
VEGFR2	Forward: 5′ CTG TAT GGA GGA AGA GGA AGT G3′	62
	Reverse: 5′ GGT TTC TCCAAT GGG ATA TC3′	58
VEGFR3	Forward: 5′ CTC TGA CCT AGT GGA GAT CCT G3′	64
	Reverse: 5′ CTT CGG TGA TAT GTA GAG CTG TG3′	63
MMP-2	Forward: 5′ AAC TTC CGA TTA TCC CAT GAT3′	55
	Reverse: 5′ GGC CAG TAC CAG TGT CAG AG3′	60
MMP-9	Forward: 5′ TGT TCC CGT TCA TCT TTG AG3′	56
	Reverse: 5′ ATC CTG GTC ATA GTT GGC TGT3′	59
MMP-14	Forward: 5′ GCT TTA CTG CCA GCG TTC3′	56
	Reverse: 5′ CCC ACT TAT GGA TGA AGC AAT3′	57
TIMP-1	Forward: 5′ CAG TAA GGC CTG TAG CTG TGC3′	63
	Reverse: 5′ AGG TGG TCT CGT TGA TTT CTG3′	59
TIMP-2	Forward: 5′ AGG TGG TCT CGT TGA GCA ACC3′	59
	Reverse: 5′ GGC CGT GTA GAT AAA CTG GAT3′	59
GAPDH	Forward: 5′ GGC CTT CCG TGT TCC TAC3′	58
	Reverse: 5′ TGT CAT CAT ACT TGG AGG TT3′	54

### 2.6 Estimation of VEGF in patients’ serum samples using ELIZA kit

Institutional human ethical clearance was obtained prior to work with the human samples (IHEC/IRB no. IHEC-0220/N/M). The NS1-positive samples of dengue were included in the study. The estimation of VEGF in the patients’ serum samples was done as per the manufacturer’s protocol (Aviva Biology). At least 50 or more positive samples were screened for VEGF; however, the data represented is the average of all four serotypes compared with the healthy control. Human VEGF ELISA Kit was used to determine the concentrations of serum VEGF factors.

### 2.7 Experiment with mice

BALB/c mice was used in the current study, and institutional animal ethical committee approval was obtained prior to begin the study (approval no. ICMR-VCRC/IAEC/2020/3/). The BALB/c mice were treated with saline intraperitonially or recombinant NS1 (obtained from R&D biological company) of serotype-2 (50 µg/mice) antigen alone or in combination with atorvastatin (40 mg/kg) two doses with a time frame of 4 days. Blood samples were collected by cardiac puncture and kept for RNA isolation. RNA isolation was done, and assessment of matrix metalloproteases and other genes was done by using real-time PCR. Gene expression was normalized with GAPDH internal control. The data presented is one of the representatives of three independent experiments performed in triplicates or not less than duplicates.

### 2.8 Statistical analysis

The data is calculated and analyzed, which is represented as mean ± SEM. The software GraphPad Prism 5 was selected for the analysis needed for statistical purposes. ANOVA test (one-way analysis of variance) was done, using Tukey’s test or Newman–Keuls test as *post-hoc* test. A *p*-value <0.05 was accepted as statistically significant.

## 3 Results

### 3.1 All four types of dengue virus (1–4) differentially regulate MMP expressions in peripheral blood mononuclear cells and in cultured THP-1 cells

MMPs may be involved in the modulation of the immune response during severe dengue. They can influence the migration and activation of immune cells, such as monocytes and macrophages, and the release of pro-inflammatory cytokines and chemokines. This dysregulated immune response, in conjunction with increased vascular permeability, can further contribute to the severity of the disease. In the present study, we have inoculated THP-1 cells with the whole virus of all four serotypes of dengue virus and studied the expression profiles of various MMPs in it.

**Figure 1:**
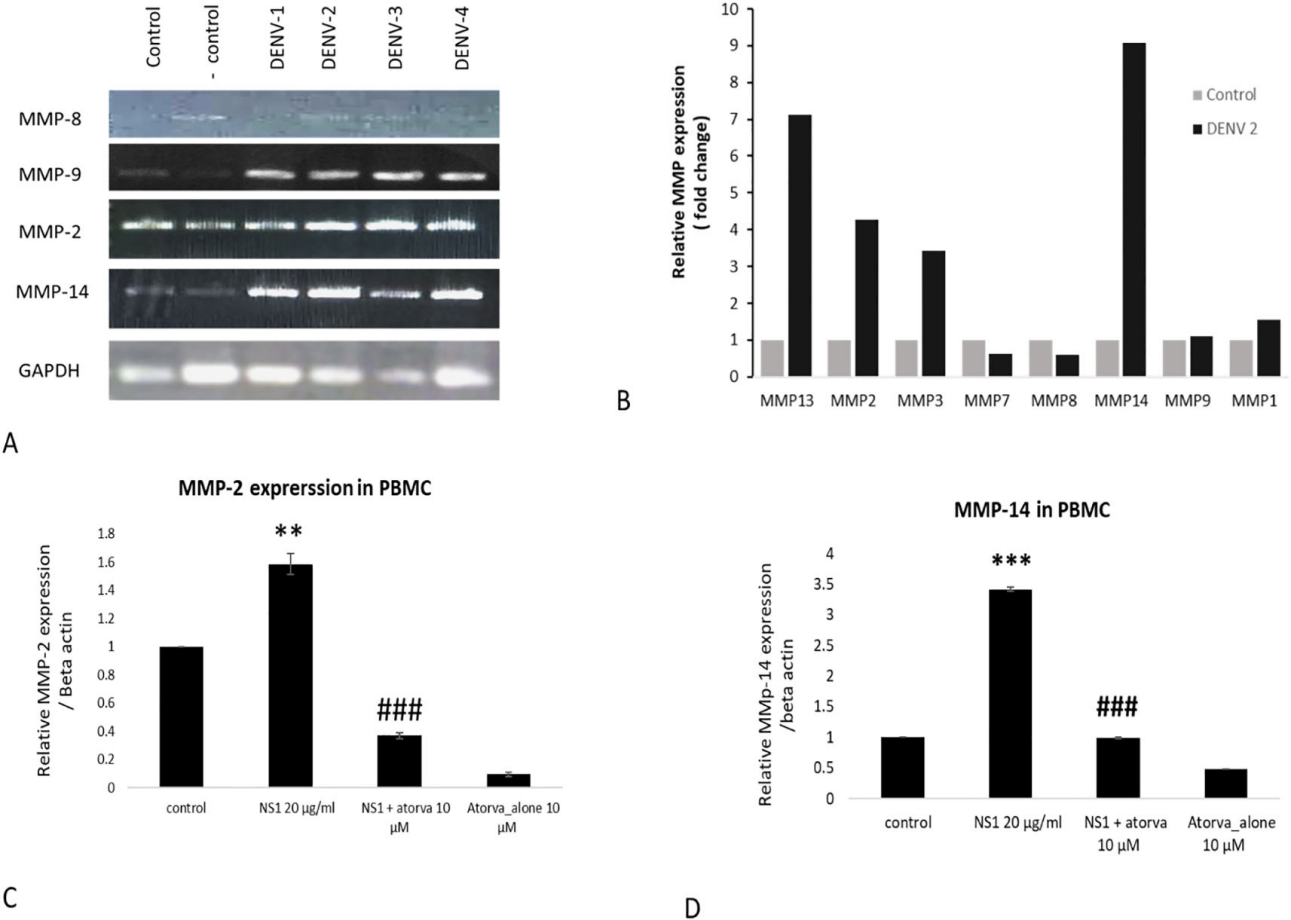
Effects of different dengue virus serotypes on the expressions of MMPs in monocyte cells. **(A)** Effects of different dengue virus serotypes on the expression profile of MMP-8, MMP-9, MMP-2, and MMP-14 in THP-1 monocyte cells. **(B)** Real-time PCR measurement on the effects of dengue virus type-2 serotype (DENV2) on the expression profile of MMPs in THP-1 monocyte cells. **(C)** Effect of atorvastatin on NS1-induced MMP-2 expression in primary PBMC. **(D)** Effect of atorvastatin on NS1-induced MMP-14 expression in primary PBMC. #≤0.05, **≤0.01 and ***p≤0.001 significant compared with control. ### p≤0.001 significant compared with NS1 treated group.

In the present study, we have found a significant increase in the *MMP-2* levels in the THP-1-infected cells with all four serotypes. A higher expression of *MMP-14* was observed in the DENV-infected group compared to the control group. Homogenous expression of the GAPDH was observed in all the treated and control groups (**Figure 1**). Similarly, a significant increase in the expression of *MMP-9* was observed in the DENV 1 to 4 serotype-infected group compared to the respective control group (**Figure 1A**). Further real-time PCR also showed a higher expression of other MMPs, i.e., MMP-13, MMP-2, MMP-3, MMP-14, MMP-9, and MMP-1, with the exposure of DENV-2 virus compared with control or unexposed THP-1 cells (**Figure 1B**). Similarly, as shown in **Figures 1C, D**, atorvastatin has significantly attenuated the MMP-2 and MMP-14 expression in NS1-induced peripheral blood mononuclear cells (PBMC).

### 3.2 Atorvastatin attenuated DENV-2 (serotype-2 of dengue virus)-induced mRNA expression profile of MMPs in THP-1, monocyte cells

**Figure 2:**
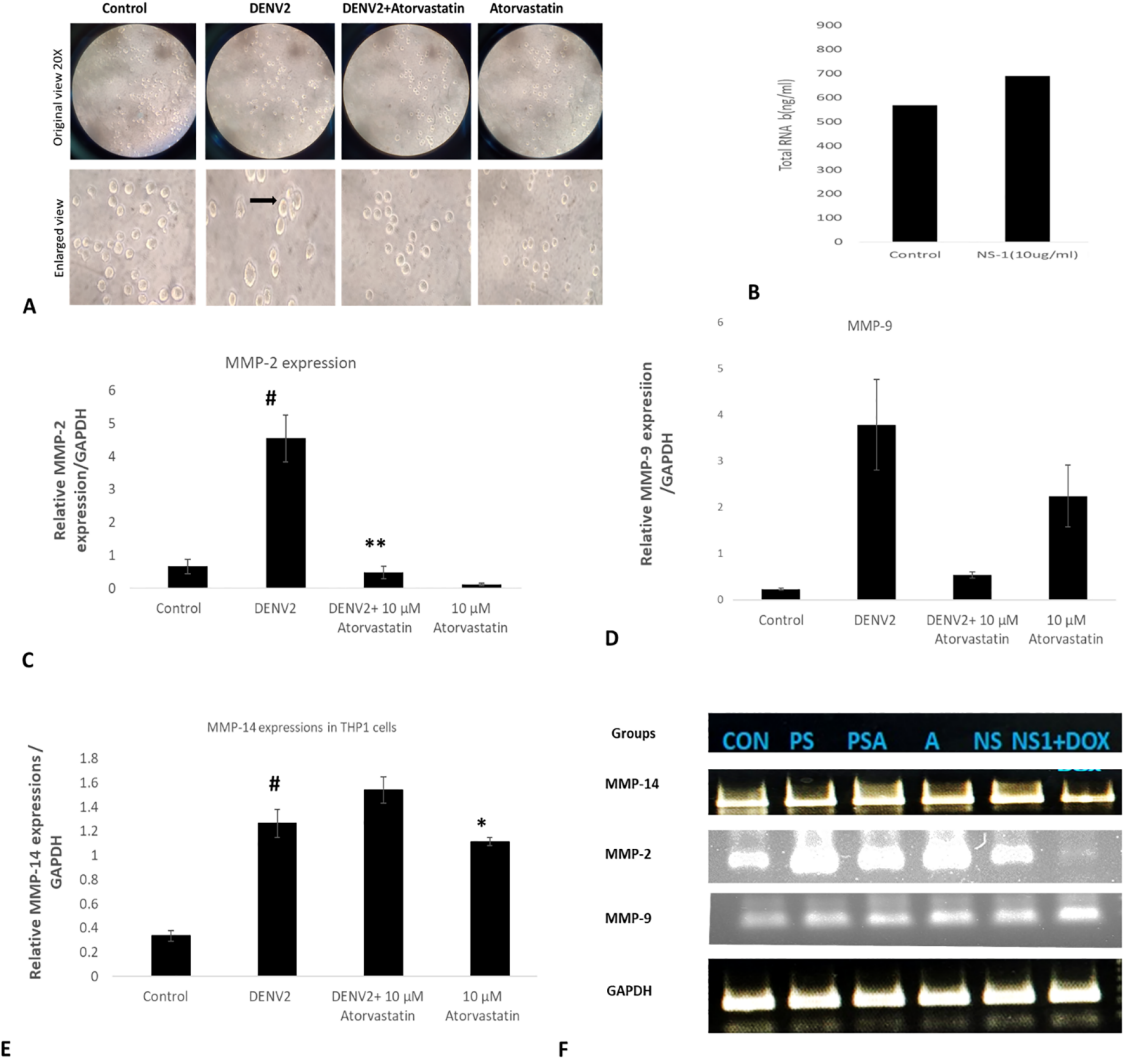
Effect of atorvastatin against DENV-2 virus in THP-1 monocyte cells. Presented in the figure is the expression of MMP genes in the DENV-2 serotype-infected THP-1 cells in the presence and absence of atorvastatin. **(A)** Morphological assessment and cytopathic effect on the THP-1 monocyte cells in the presence and absence of atorvastatin. **(B)** Total transcriptome (total RNA in the presence and absence of DEN2 virus. **(C)** Expression of MMP-2 genes in the DENV-2 serotype-infected THP-1 cells in the presence and absence of atorvastatin. **(D)** Expression of MMP-9 genes in the DENV-2 serotype-infected THP-1 cells in the presence and absence of atorvastatin. **(E)** Relative mRNA expression/GAPDH of *MMP-14* gene in THP-1 cells infected with DENV-2 serotype. **(F)** Final quantitative PCR product run in the agarose gel. #<0.05 significant compared with control. *<0.05, **<0.01 significant compared with NS1 treated group.

Next, we have inoculated THP-1 cells with the whole DENV-2 serotype of dengue virus with or without atorvastatin (drug) and studied the expression profiles of MMP-2, MMP-8, and MMP-14. We observed a change in the morphology of THP-1 cells as well as in the total RNA content (transcriptome) of the cells in response to dengue virus (**Figures 2A, B**). We observed a significant increase in the *MMP-2* levels in THP-1 infected with DENV-2 compared to the control group (without DENV-2 infection) as well as the other groups; a significant decrease in the expression levels of *MMP-2* was also seen in the DENV-2 + atorvastatin-infected group and only-atorvastatin-treated group (**Figure 2C**). A higher expression of *MMP-14* was observed in the DENV-2-infected group compared to the control group. However, a lower level of *MMP-14* was also observed in the DENV-2 + atorvastatin-treated group compared to the DENV-2-infected group and atorvastatin-treated group (**Figure 2E**). A homogenous expression of GAPDH was observed in all of the treated and control groups (**Figure 2F**). Similarly, a significant increase in the expression of *MMP-9* was observed in the DENV-2-infected group compared to the respective control group (**Figure 2D**). A significant decrease in the expression of *MMP-9* was observed in the DENV-2 + atorvastatin and only-atorvastatin-treated group compared to the DENV-2-infected group (**Figure 2**).

### 3.3 Atorvastatin modulated the NS1-induced mRNA expression profile of MMPs and VEGF in mice

**Figure 3:**
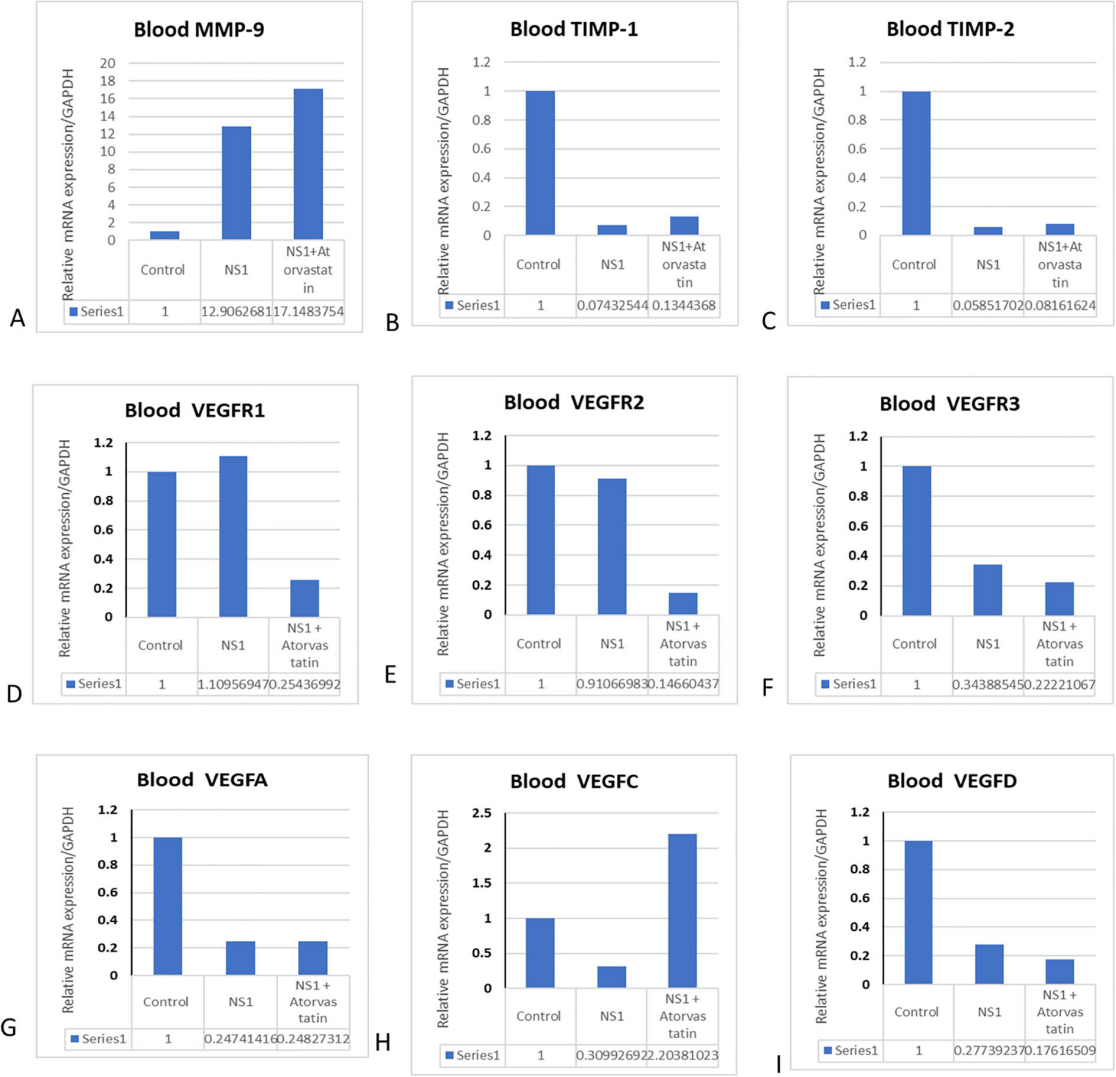
Effect of atorvastatin on matrix metalloproteinases and VEGF expressions in mice. The mice were injected with NS1 alone and in combination with atorvastatin, and different MRNA gene profile was checked in the blood. **(A)** Histograms represent the expression profile of MMP-9 in blood. **(B)** Histograms represent the expression profile of TIMP-1. **(C)** Histograms represent the expression profile of TIMP-2. **(D)** Histograms represent the expression profile of VEGFR1. **(E)** Histograms represent the expression profile of VEGF-2. **(F)** Histograms represent the expression profile of VEGFR3. **(G)** Histograms represent the expression profile of VEGF-A. **(H)** Histograms represent the expression profile of VEGF-C. **(I)** Histograms represent the expression profile of VEGF-D in blood.

In addition to the *in vitro* cultured cells, we have also checked the expression profile of MMPs in *in vivo* mouse models, and the effects of atorvastatin were assessed. We found that the expression of MMPs and VEGF is significantly increased in the mice treated with purified NS1 antigen. We have also obtained the expression of VEGF and VEGF receptors in response to NS1 alone and in combination with atorvastatin. A homogenous expression of GAPDH was observed in all of the treated and control groups (**Figure 3**). We found that NS1 treatment has significantly upregulated the MMP-9 expression and downregulated the TIMP-1 and TIMP-2 expressions in mice. Atorvastatin has significantly reverted the TIMP expressions but further potentiated the MMP-9 expressions (please see **Figures 3A–C**). It was interesting to note that VEGF family members, like VEGF-A, VEGF-C, and VEGF-D, were downregulated, and atorvastatin reversed the VEGF-C expressions (please see **Figures 3G–I**). Receptors like VEGFR2 and VEGF-R3 were also significantly downregulated, which were further downregulated by atorvastatin. However, VEGF-R1 was not significantly upregulated by the NS1 treatment to the mice (please see **Figures 3D–F**). VEGF-C was increased, while VEGFA and VEGF-D were decreased by NS1 treatment in mice (please see **Figures 3G–I**).

### 3.4 Effects of secretome obtained from DENV-2-exposed THP-1 cells on VEGFs and VEGF receptor expressions in HUVEC/endothelial cells

**Figure 4:**
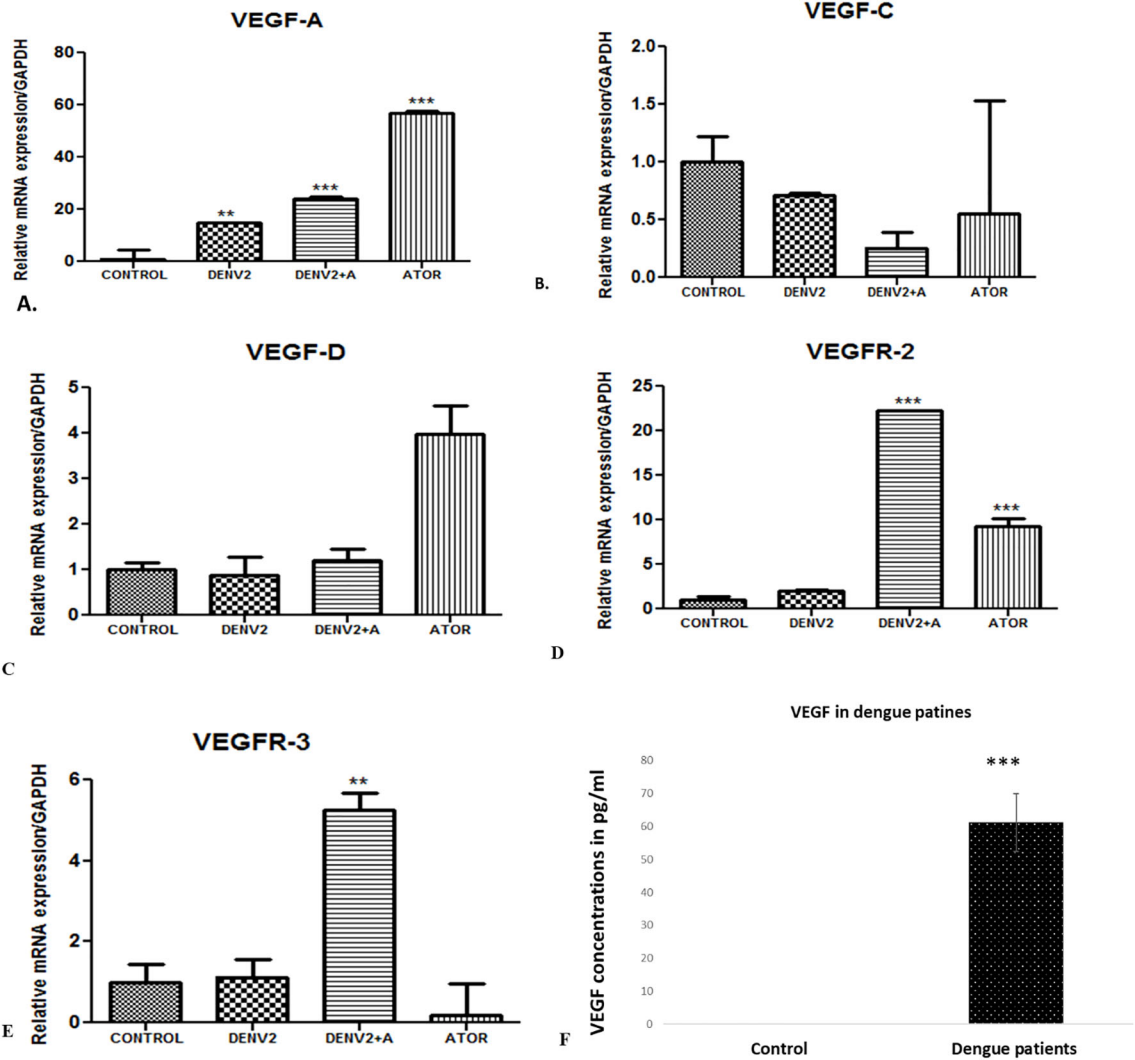
VEGF concentration in dengue patients and expression profile of genes in the *in vitro* cultured endothelial cells (HUVEC) cells exposed to supernatants obtained from DENV-2 serotype-induced THP-1 cells. **(A)** Histograms represent the relative mRNA expression/GAPDH of VEGF-A gene in HUVEC cells. **<0.01 and ***p<0.001 significant compared with control. **(B)** Expression of VEGF-C gene in the endothelial cells (HUVEC) cells exposed to supernatants obtained from DENV-2 serotype-induced THP-1 cells in the presence and absence of atorvastatin. mRNA expression of VEGF-D gene in the endothelial cells (HUVEC) cells exposed to supernatants obtained from DENV-2 serotypes-induced THP-1 cells in the presence and absence of atorvastatin. **(C)** Histograms represent the relative mRNA expression/GAPDH of VEGF-D gene in HUVEC cells. **(D)** Histograms represent the relative mRNA expression/GAPDH of *VEGFR2* gene in HUVEC cells. ***p<0.001 significant compared with dengue and dengue + A. ***p<0.001 significant compared with control and Atorvastatin. **(E)** Histograms represent the relative mRNA expression/GAPDH of *VEGFR3* gene in HUVEC cells. **p<0.001 significant compared with dengue and dengue + A. **(F)** VEGF concentration in healthy and dengue patients. ***p<0.001 significant compared with control.

Angiogenesis is characterized by the formation of new blood vessels from pre-existing ones. It is a fundamental physiological process involved in various stages of development, wound healing, and tissue repair. Angiogenesis initiates with a stimulus that triggers the need for new blood vessels, such as tissue injury or the demand for increased blood supply. There is activation of endothelial cells, which are the cells lining the inner surface of blood vessels. During angiogenesis, endothelial cells undergo proliferation, migration, and remodeling to form new blood vessels. VEGF or vascular endothelial growth factor is a pro-angiogenic factor that stimulates the formation of new blood vessels. We have observed the highest angiopoetin-1 gene expression in the DENV-2-infected group compared to the other infected groups. Downregulation in the level of angiopoetin-1 was observed in the atorvastatin-treated group (**Figure 4**). We have observed increased angiopoietin-2 levels in the group where HUVEC cells were treated with DENV-2 + atorvastatin compared to all of the other treated groups (**Figure 4**).

VEGF-A is a key pro-angiogenic factor that plays a central role in stimulating the formation of new blood vessels. We have observed upregulation of the *VEGF-A* gene in the DENV-2-infected group compared to the control group, whereas the atorvastatin-treated group expressed higher levels of *VEGF-A* compared to all of the other groups (**Figure 4A**). Downregulation in the level of *VEGF-C* was seen in the DENV-2 + atorvastatin-treated group compared to DENV-2-treated group (**Figure 4B**). In addition, we have observed VEFG-C downregulation in all of the treated groups compared to the control group (**Figure 4B**).

VEGF-D is known to be involved in regulating vascular permeability and angiogenesis. Some research have suggested that, in dengue infection, the levels of VEGF-A and VEGF-C may be altered, contributing to the increased vascular permeability seen in severe cases. However, the specific role of VEGF-D in dengue infection has not been well documented. In the present study, we found no significant difference in VEGF-D expression in the DENV-2-infected group or DENV-2 + atorvastatin-treated group compared to the control group (**Figure 4C**).

VEGFRs are a family of receptors that specifically bind to and mediate the effects of vascular endothelial growth factors (VEGFs), a group of proteins involved in angiogenesis and vascular development. VEGFR-2 is the primary receptor responsible for mediating the angiogenic effects of VEGF-A. It plays a crucial role in promoting endothelial cell proliferation, migration, and survival, leading to the formation of new blood vessels. We have observed upregulation of the *VEGFR-2* gene in the DENV-2 + atorvastatin-infected group and atorvastatin-infected group, whereas DENV-2 + atorvastatin-treated group expressed higher levels of *VEGFR-2* compared to all of the other groups (**Figure 4D**). VEGFR-3 is primarily involved in the formation of new lymphatic vessels. It is the receptor for VEGF-C and VEGF-D. DENV-2 + atorvastatin-treated group expressed higher levels of *VEGFR-3* compared to all of the other groups (**Figure 4E**). Furthermore, we found that VEGF protein concentration was increased in the serum samples of dengue NS1-positive samples, supporting the other results for a mechanism point of view (**Figure 4F**).

### 3.5 Secretome obtained from DENV-2-exposed THP-1 cells altered the angiogenic and apoptotic parameters in HUVEC/endothelial cells

To further assess the THP-1 monocyte-mediated effects on endothelial cells, we have exposed the THP-1 monocyte cells to DENV-2 virus alone and in combination with atorvastatin and collected the supernatants after 24 h of exposure. These superannuates from THP-1 cells (secretome) were exposed to human endothelial HUVEC cells for 24 h, and their apoptosis and/or angiogenesis parameters were measured.

**Figure 5:**
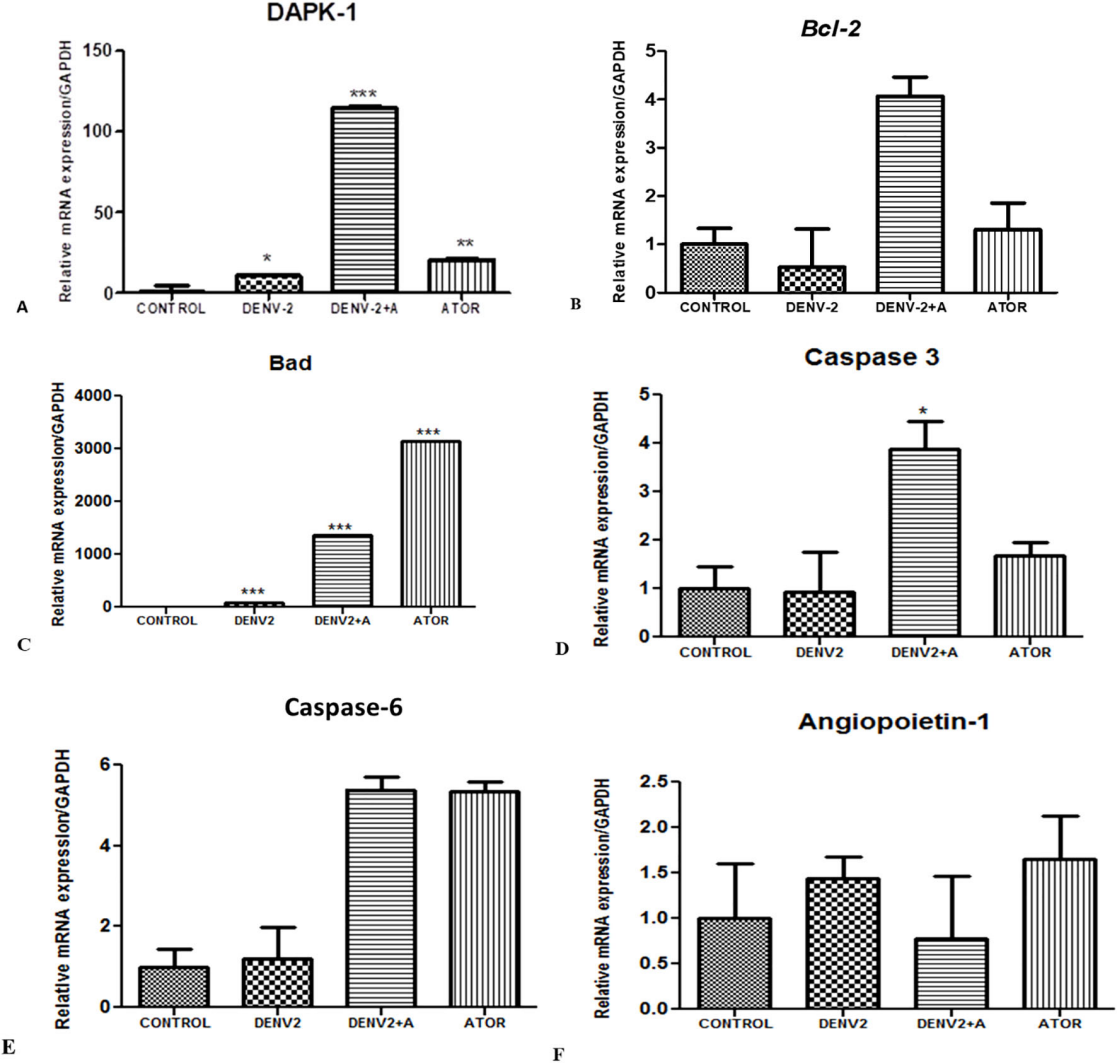
The assessment of apoptosis in endothelial cells (HUVEC) cells exposed to supernatants obtained from DENV-2-induced THP-1 cells. **(A)** Relative mRNA expression/GAPDH of *DAPK-1* gene in HUVEC cells infected with DENV-2 serotype. *<0.05, significant compared with control and DENV-2. ***p<0.001 significant compared with dengue-2 and dengue + A. F. **p<0.001 significant compared with control and ATOR (atorvastatin). **(B)** Relative mRNA expression/GAPDH of *Bcl-2* gene in HUVEC cells exposed to supernatants from THP-1 cells. **(C)** Relative mRNA expression/GAPDH of *BaD* gene in HUVEC cells treated with the supernatants of THP-1 monocytes. ***p<0.001 significant compared with control and DENV-2. ***p<0.001 significant compared with dengue-2 and dengue + A. ***p<0.001 significant compared with control and ATOR (atorvastatin). **(D)** Relative mRNA expression/GAPDH of *Caspase-3* gene in HUVEC cells. *P<0.05 significant compared with dengue-2 and dengue-2+ A. **(E)** Histograms represent the relative mRNA expression/GAPDH of *Caspase-6* gene in HUVEC cells. **(F)** Histograms represent the relative mRNA expression/GAPDH of angiopoietin-1 gene in HUVEC cells.

Apoptosis is a highly regulated cell death process in a highly programmed manner to eliminate damaged, neoplastic, and virus-infected cells. The Bcl-2 family protein regulates the apoptosis program; these proteins either promote or inhibit the apoptosis process, such as Bcl-2 proteins (anti-apoptotic); Bax, Bad, and Bak are pro-apoptotic. When exposed to supernatants obtained from DENV-2-exposed THP-1 cells, HUVEC/endothelial cells showed increased levels of mRNA expression compared to the control (**Figure 5**).

DAPK-1 stands for death-associated protein kinase 1. It is involved in regulating cell death, apoptosis, and autophagy. DAPK-1 is known to play important roles in immune responses. DAPK-1 is activated in response to DNA damage, oxidative stress, and pro-inflammatory cytokines. Once activated, DAPK-1 can phosphorylate and activate Bax, leading to the activation of apoptotic pathways. We have found increased *DAPK-1* levels in the DENV-2-infected group compared to the control, whereas significantly higher *DAPK-1* levels were observed in the DENV2 + atorvastatin group compared to all of the other groups (**Figure 5A**).

Bcl-2 is anti-apoptotic, which promotes cell survival and inhibits apoptosis. It prevents cytochrome c release from the mitochondria. We have also observed downregulation of the *Bcl-2* gene in the DENV-2-infected group compared to the control group, whereas DENV-2 + atorvastatin and atorvastatin-treated group expressed higher levels of *Bcl-2* compared to the control group and the DENV-2-infected group (**Figure 5B**).

BAD is a pro-apoptotic protein involved in promoting apoptosis by antagonizing the effects of anti-apoptotic Bcl-2. Dephosphorylated Bad binds to Bcl-2 and prevents its protective effects. This allows Bax and Bak to trigger mitochondrial cytochrome c release and initiates the apoptotic process. We have observed upregulation of the *Bad* gene in the DENV-2-infected group compared to the control group, whereas the atorvastatin-treated group expressed higher levels of *Bad* compared to all of the other groups in the HUVEC/endothelial cells exposed to the supernatant from DENV2-exposed THP-1 cells (**Figure 5C**).

Caspase-3 is an effector or executioner caspase. It carries out the final steps of apoptosis. Once activated, it cleaves and activates other caspases. Caspase-3 is typically activated through proteolytic processing by initiator caspases, such as caspase-8 or caspase-9, which are triggered by specific apoptotic signals. In the present study, we have observed a significantly higher caspase-3 expression in the DENV-2 + atorvastatin-treated group compared to all of the other infected groups. The atorvastatin-treated group also showed higher levels of caspase-3 in the control group and DENV-2-infected group (**Figure 5D**). Similar to the expression pattern observed for caspase-6, significantly high levels were observed in DENV-2 + atorvastatin- and atorvastatin-treated groups compared to all of the other groups (**Figure 5E**). It is important to notice that angiopoietin-1 was also reversed by atorvastatin (**Figure 5F**).

### 3.6 Purified MMP-9 also affected the angiogenic, apoptotic, and VEGF parameter in HUVEC/endothelial cells in a similar fashion

**Figure 6:**
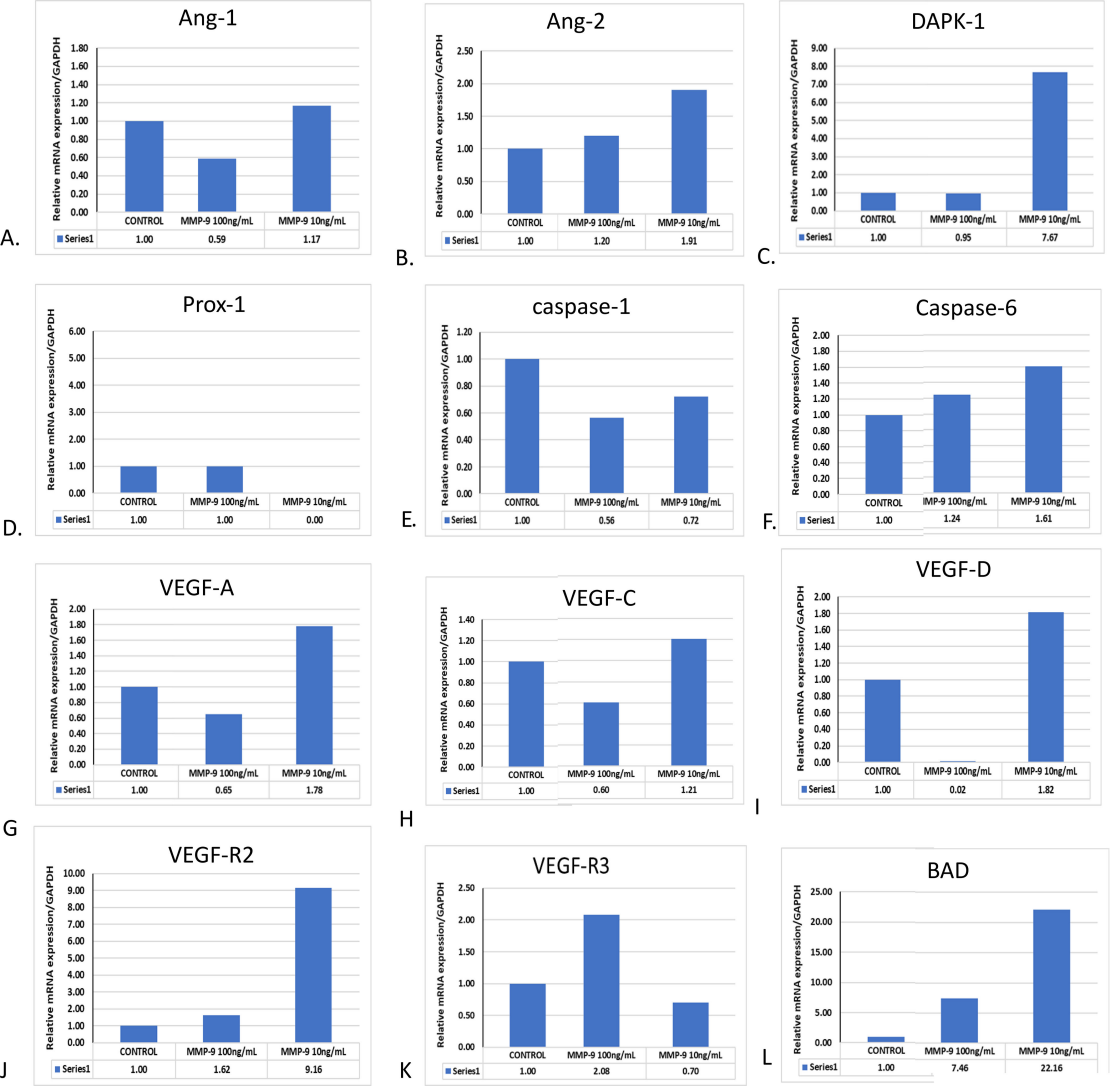
Effect of purified MMP-9 on the angiogenic, apoptotic, and VEGF expression profiles in HUVEC/endothelial cells. HUVEC cells were treated with purified MMP-9 alone in different concentrations, and the measurement of mRNA expression profile was done. **(A)** Histograms represent the expression profile of angiopoietin-1. **(B)** Histograms represent the expression profile of angiopoietin-2. **(C)** Histograms represent the expression profile of DAPK-1. **(D)** Histograms represent the expression profile of Prox-1. **(E)** Histograms represent the expression profile of caspage-1. **(F)** Histograms represent the expression profile of caspase-6. **(G)** Histograms represent the expression profile of VEGF-A. **(H)** Histograms represent the expression profile of VEGF-C. **(I)** Histograms represent the expression profile of VEGF-D in blood. **(J)** Histograms represent the expression profile of VEGF R2. **(K)** Histograms represent the expression profile of VEGFR3. **(L)** Histograms represent the expression profile of Bad.

In our previous cell culture-based experiments and in the existing literature, we found that NS1 exposure and DENV-2 significantly upregulated the MMP-9 expression; however, its mechanism was not properly understood. We speculated that MMP-9 overexpression might have caused vasculature damage by one or many mechanisms. Just to test this hypothesis, we have exposed different concentrations of MMP-9 on *in vitro* cultured HUVEC/endothelial cells. We found that the expression profiles of angiopoietin-1, angiopoietin-2, and DAPK-1 were upregulated in HUVEC cells, while prox-1 and caspage-3 were significantly downregulated (please see **Figures 6A–E**). Apoptotic gene caspase-6 was also upregulated (**Figure 6F**). VEGF family members like VEGF-A, VEGF-C, VEGF-D, and their receptors like VEGFR2 and VEGF-R3 were also significantly increased with a low dose of 10 ng/mL in 24 h of exposure time (**Figures 6G–I**). The similar result were also obtained for VEGF R2, R3 & BAD (**Figures 6J–L**).

## 4 Discussion

**Figure 7:**
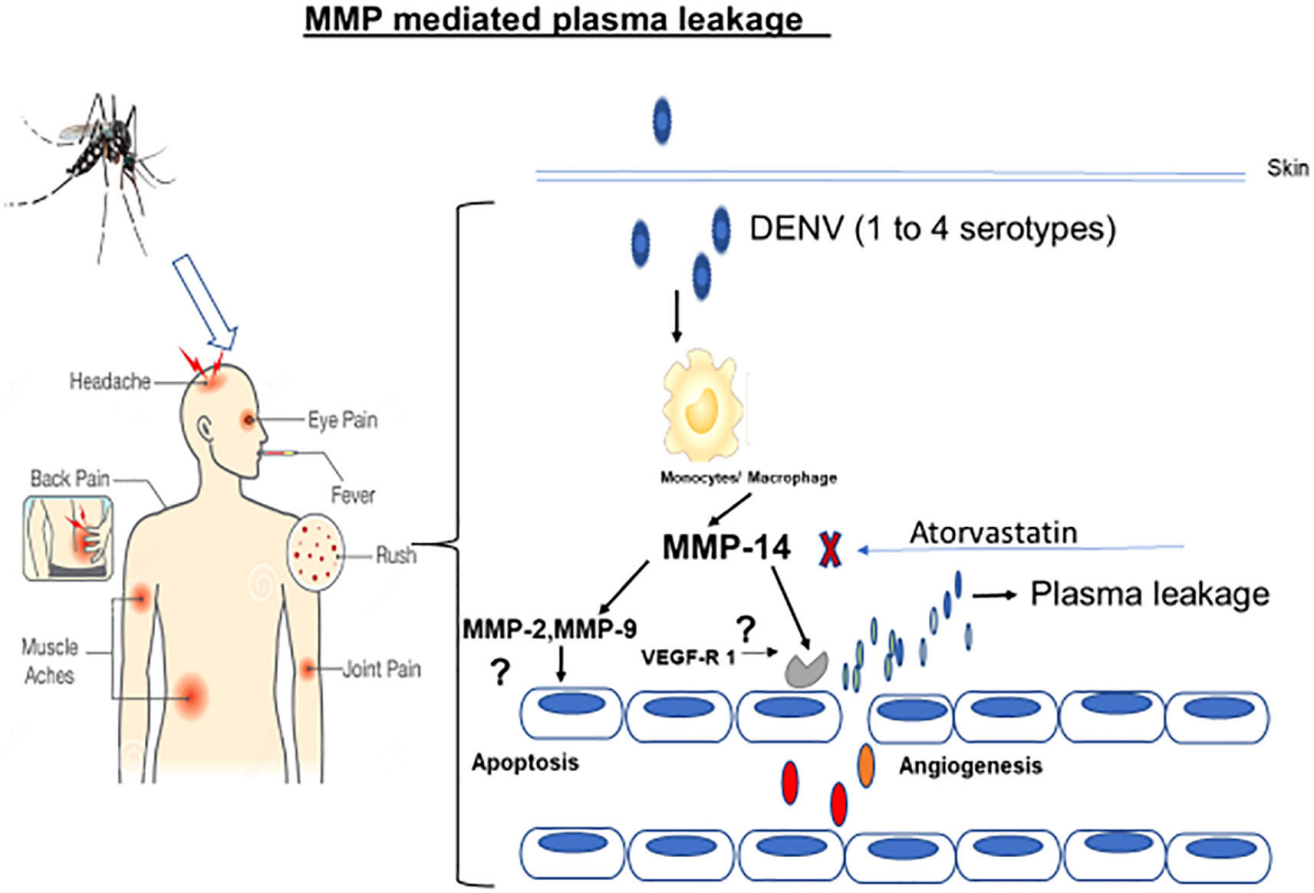
Diagrammatic representation of the mechanism of action of dengue virus-induced monocyte-mediated endothelial dysfunctions. As shown in the diagram, the dengue virus potentially induces monocytes to upregulate MMPs’ expressions which later interact with the endothelial cells to cause the endothelial dysfunctions leading to dengue shock syndrome.

In the present study, we report that the expression profiles of MMPs in primary monocytes and in THP-1 cells are highly upregulated when they are exposed to all four different dengue serotypes (**Figure 7**). We showed that MMP-2, MMP-9, and MMP-14 in the DENV virus-exposed THP-1 cells were highly upregulated when compared to the unexposed control cells. Interestingly, these upregulated expressions of MMPs were significantly reversed by atorvastatin. Atorvastatin exerted a similar kind of effects in *in vivo* mouse models when injected along with the NS1 antigen of dengue virus type-2 serotype. The mechanism of monocyte-mediated endothelial dysfunctions was revealed by exposing secretome obtained from dengue virus-induced monocytes. The highly upregulated expressions of pro-apoptotic markers caspases, angiopoietins, and their receptor genes were found in the DENV-2-induced group compared to the control in endothelial (HUVEC) cells. The refined mechanism of immunopathogenesis due to MMP-9 was unveiled on the endothelial cells.

Different serotypes and genotypes of the dengue virus (DENV) do play a unique role in causing varying levels of pathology in dengue viral disease, particularly in the development of dengue hemorrhagic fever (DHF) and dengue shock syndrome (DSS) (Afreen et al., 2014; Acosta-Perez et al., 2022). Furthermore, within each serotype, there can be multiple genotypes or strains of the virus circulating in different regions (Afreen et al., 2014; Ahmad and Poh, 2019; Adikari et al., 2020). These genotypes can differ in their genetic makeup and virulence, contributing to variations in disease severity (Arayasongsak et al., 2020). Certain genotypes of DENV have been associated with more severe forms of the disease, while others may cause milder symptoms (Arayasongsak et al., 2020; Adams et al., 2024). The interactions between the host immune response, viral factors, and the specific serotypes and genotypes of DENV are complex and not yet fully understood (Abdul Rahman et al., 2024; A et al., 2024). Understanding the role of serotypes and genotypes in dengue pathology is crucial for disease surveillance, vaccine development, and targeted interventions (Moragas et al., 2023; Akram et al., 2024).

MMPs have been implicated in dengue viral infection and are suspected to contribute to plasma leakage, which can lead to severe manifestations such as DHF/DSS (Her et al., 2017). MMPs are involved in the degradation and remodeling of extracellular matrix components (Voraphani et al., 2010). We hereby report elevated MMP-2, MMP-9, and MMP-14 in the DENV-2-infected THP-1 cells compared to an uninfected control group (Moragas et al., 2023). In addition to this, we have also treated THP-1 cells with atorvastatin drug before DENV-2 serotype infection and a combination of both DENV-2 + atorvastatin drug. We found significantly low levels of MMPs in the combination group of DENV-2 + atorvastatin (Lien et al., 2024). This suggested that dengue virus also causes similar effects to that of its purified NS1 antigen and that atorvastatin shows an anti-dengue virus effect. The current finding is also in the line with our previous publications from the labs showing the purified NS1 antigen’s effect on monocyte cells (Niranjan et al., 2019b; Niranjan et al., 2019a).

The monocyte-mediated matrix metalloproteinases may cause the cell death of endothelial cells by apoptosis (Catteau et al., 2003; Auerswald et al., 2021; Lien et al., 2024). Antiapoptotic mediator Bcl-XL can delay apoptosis in DEN virus-infected N18 cells without inhibiting virus replication (Catteau et al., 2003; Romero-Cruz et al., 2024). Bcl-XL is a member of the Bcl-2 family of proteins, which are known regulators of apoptosis. Bcl-XL is predominantly expressed in the nervous system and has been shown to have antiapoptotic properties (Catteau et al., 2003). By overexpressing Bcl-XL in DEN virus-infected N18 cells, researchers observed a delay in the occurrence of apoptosis. This suggests that Bcl-XL can provide protection against apoptosis induced by DEN virus infection, allowing the infected cells to survive for a longer period (Decotter et al., 2023). On the other hand, the proapoptotic regulator Bcl-XS, which is another isoform of the Bcl-X protein, was found to potentiate DEN-2 virus-induced apoptosis in the same study (Su et al., 2001). We have observed an elevated expression of pro-apoptotic Bax and DAPK-1 in the DENV-2 + atorvastatin-treated group compared to the control, DENV-2-infected THP-1, and atorvastatin treated THP-1 groups (Lien et al., 2024; Wilson and McCormick, 2024). Higher levels of caspase-3 and caspase-6 were seen in the DENV-2 + atorvastatin-treated group compared to the control, DENV-2-infected THP-1, and atorvastatin-treated THP-1 groups (Moragas et al., 2023; Pagliari et al., 2024). These results indicated that the one with atorvastatin significantly reduced the MMP expression but also induced the apoptotic effects of endothelial cells (Gam et al., 2021).

It is believed that VEGF plays very important roles in the maintenance of vasculature and other endothelial cells’ homeostasis (Niranjan et al., 2019; Li et al., 2022; Lim et al., 2024). A recent study also shows that there is a significant role of VEGFs in the pathogenesis of severe dengue viral disease (Gam et al., 2021; Lim et al., 2024). The response of endothelial cells to angiogenic mediators is also important in studying vascular leakage (Annan et al., 2023; Apoorva et al., 2024). Short-lived plasma leakage is a characteristic feature of severe dengue (DENV) disease (Niranjan et al., 2019; Lien et al., 2024). The breakdown of the endothelial barrier is believed to play a role in local vascular leakage. *In vitro* studies have shown that endothelial cells can be infected by DENV virus, and interestingly, infection with DENV-2 virus has been found to induce apoptosis in HUVEC (Guntamadugu et al., 2024). In the present study, angiopoietins and their receptor genes, such as Ang-2, VEGFR-2, and VEGFR-3, were highly expressed in the DENV-2 + atorvastatin-treated group compared to the other groups. DENV virus infection has been shown to activate nuclear factor-kappa B (NF-κB) in human hepatocytes, neurons, and endothelial cells (Nanaware et al., 2021). NF-κB is a transcription factor that plays a crucial role in the alteration of immune responses, inflammation, and cell survival (Salgado et al., 2023). Angiopoietin-1 is important in maintaining the endothelial cell barrier integrity, and angiopoietin-2 is associated with endothelial permeability and is elevated in severe plasma leakage (Pagliari et al., 2024). Increased levels of angiopoietin-1 and angiopoietin-2 in the presence of DENV-2 serotype were observed. Furthermore, the altered expression profile of VEGF growth factors and their receptors provides an important mechanism of immune cell-mediated regulation of endothelial cells in dengue infection (Baraya et al., 2019). As shown in the results, the DENV-2 serotype may be secreting high levels of VEGF growth factors which will be exerting their effects on endothelial cells, making them dysfunctional. We found that DENV type 2 infections could mediate hyperpermeability (Bonanomi and Lavezzo, 2013; Henrique Ferreira Sucupira et al., 2023).

MMP-9 is believed to be terminal molecules responsible for the damage to endothelial cells by one of the many mechanisms along with other MMPs (Khalilpour et al., 2019; Wang et al., 2019). It is evident in the literature that MMP-9 participates in neutrophil-mediated endothelial dysfunction in systemic lupus erythematosus disease (Carmona-Rivera et al., 2015). Matrix metalloproteinase-1 and matrix metalloproteinase-9 were found to regulate endothelial cell-mediated functions (Davis et al., 2001). In our study, we have observed that MMP-9 has significantly upregulated angiopoietin-1, angiopoietin-2, and DAPK-1 expressions, suggesting the angiogenesis and apoptosis of endothelial cells (Makgoo et al., 2023). DAPK-1 is a tumor suppression gene and induces apoptosis in several cells associated in various diseases. Furthermore, matrix metalloproteinase-9 was involved in the causation of endothelial dysfunction in atherosclerosis via protease-activated receptor-1-mediated mechanism (Davis et al., 2001; Florence et al., 2017). MMP-9 expression and activity were found to be concurrent with endothelial cell apoptosis, providing a major relevant evidence in rat models of basilar artery after subarachnoid hemorrhaging (Guo et al., 2015). This study provided a strong support for the role of MMP-9 in causing dengue hemorrhagic fever in severe dengue viral disease. It is further important to note that, in the present study, MMP-9 has significantly upregulated the VEGF members and their receptors’ expressions, suggesting their mechanism of angiogenesis (Adya et al., 2008; Chen et al., 2024). In a study, it was found that visfatin produced VEGF and MMP-2/9 production via MAPK and PI3K/Akt signaling pathways causing angiogenesis (Adya et al., 2008). The present findings on the effect of MMP-9 on endothelial cells show that it causes angiogenesis and apoptosis, affecting vascular permeability and thus resembling the immunopathogenesis of dengue viral disease.

Collectively, in conclusion, this study provides new insights, i.e., DENV-2 activates monocytes for overexpression and the release of matrix metalloproteinases which, in turn, causes angiogenesis and apoptosis of endothelial cells, making vascular dysfunctions, which may resemble the mechanism of pathogenesis of dengue shock syndrome (DHF/DSS). Additionally, our finding shows the antiviral potential of atorvastatin which may be adopted in clinical trials against severe dengue viral disease. The current findings are interesting. However, further experiments are needed to validate the results in the future.

## Data Availability

The datasets presented in this article are not readily available because these may be required for IPR or programme issues. Requests to access the datasets should be directed to riturajniranjan@rediffmail.com.
